# Reshaping preoperative treatment of pancreatic cancer in the era of precision medicine

**DOI:** 10.1016/j.annonc.2020.11.013

**Published:** 2021-02

**Authors:** R. Casolino, C. Braconi, G. Malleo, S. Paiella, C. Bassi, M. Milella, S.B. Dreyer, F.E.M. Froeling, D.K. Chang, A.V. Biankin, T. Golan

**Affiliations:** 1 Wolfson Wohl Cancer Research Centre, Institute of Cancer Sciences, University of Glasgow, Bearsden, Glasgow, Scotland, UK; 2 Department of Medicine, University and Hospital Trust of Verona, Verona, Italy; 3 Department of Surgery, University and Hospital Trust of Verona, Verona, Italy; 4 Department of Medicine, Medical Oncology, University and Hospital Trust of Verona, Verona (VR), Italy; 5 West of Scotland Pancreatic Unit, Glasgow Royal Infirmary, Glasgow, UK; 6 Edinburgh Cancer Centre, Western General Hospital, NHS Lothian, Edinburgh, UK; 7 South Western Sydney Clinical School, Faculty of Medicine, University of NSW, Liverpool, NSW, Australia; 8 Oncology Institute, Sheba Medical Center, Tel Hashomer, Israel

**Keywords:** Pancreatic cancer, preoperative, neoadjuvant, precision medicine, prognostic biomarkers, predictive biomarkers

## Abstract

This review summarises the recent evidence on preoperative therapeutic strategies in pancreatic cancer and discusses the rationale for an imminent need for a personalised therapeutic approach in non-metastatic disease. The molecular diversity of pancreatic cancer and its influence on prognosis and treatment response, combined with the failure of ‘all-comer’ treatments to significantly impact on patient outcomes, requires a paradigm shift towards a genomic-driven approach. This is particularly important in the preoperative, potentially curable setting, where a personalised treatment allocation has the substantial potential to reduce pancreatic cancer mortality.

## Highlights

• Molecular diversity of pancreatic cancer requires a paradigm shift towards a genomic-driven therapeutic approach.
• Unselected treatment strategies demonstrate only limited efficacy in early-stage pancreatic cancer.
• Personalised treatment in non-metastatic disease has potential to reduce pancreatic cancer mortality.
• It is fundamental to implement preoperative clinical studies enriched for potential prognostic/predictive biomarkers.
• Novel models of therapeutic development are warranted to accelerate progress in pancreatic cancer care and research.

## Introduction

Pancreatic cancer (PC) is one of the most lethal solid malignancies and is predicted to soon become the second leading cause of cancer mortality in developed countries.^1^ Estimates of temporal trends for PC incidence and mortality produced by GLOBOCAN 2018 indicate a worldwide trend towards a dramatic increase of both incidence (+77.7% with 356 358 new cases) and mortality (+79.9%, 345 181 deaths) from 2018 to 2040.^2^ This is mostly due to our inability to improve prevention and treatment approaches, despite major efforts in preclinical and clinical research that have marginally impacted patient outcomes over the past 50 years. In fact, this incremental progress translates to an increased 5-year survival rate from 6% to only 9% in the years 2014-2018, resulting in a mortality/incidence ratio of 94%.^2^ There is indeed an urgent need to reduce both PC incidence, by implementing research on primary and secondary prevention, and mortality, by accelerating therapeutic development.

Surgery with radical intent represents the only potential curative treatment option for PC patients; however, only 20% of cases are diagnosed with anatomically resectable disease.^3^ Notwithstanding substantial improvement in surgical techniques and post-operative outcomes, the overall recurrence rate after resection is approximately 85% and the 5-year survival less than 30%.^4^, ^5^, ^6^, ^7^ The best adjuvant chemotherapy regimen (modified FOLFIRINOX, i.e. 5-fluorouracil, leucovorin, oxaliplatin, and irinotecan) likely adds modest survival benefit in all-comers at the expense of a considerable toxicity.^7^ The reasons for these poor outcomes stem from the inherent aggressiveness of PC, that lends it to be defined as ‘metastatic ab-initio’ disease, irrespective of the clinical stage. In fact, up to 26% of patients are found with occult metastases during surgical exploration,^8^ and approximately 70% of resected cases have nodal involvement on pathology after surgery.^9^ Furthermore, despite the importance of adjuvant therapy, studies demonstrated that up to 45% of patients are not able to receive the treatment after resection due to poor performance status, post-operative morbidity, or early progression of disease.^10^^,^^11^ Besides, fully completed adjuvant chemotherapy is an independent prognostic factor for survival after resection; however, only 55%-75% of those who initiate adjuvant therapy complete the treatment.^12^ In this context, increasing interest has been driven towards primary systemic treatments, initially investigated in borderline resectable and locally advanced PC with induction/cytoreductive intent^13^^,^^14^ and, more recently, applied to patients with resectable disease as a pure neoadjuvant (NAT) strategy.^15^^,^^16^ Preoperative treatment has been associated with several potential benefits including *in-vivo* chemosensitivity test, tumour shrinking with decreased nodal involvement, increased margin-negative resection rates, early treatment of occult micrometastases, improved compliance with chemotherapy, improved survival after curative resection, and better selection of patients who are more likely to benefit from surgery.^17^, ^18^, ^19^, ^20^ However, the role of NAT in PC is still debated due to a relative lack of robust clinical trial data supporting this approach.^21^, ^22^, ^23^ Particularly, several barriers have limited its application and data interpretation including the low response rate to chemotherapy in metastatic disease, the difficulty in assessing the impact of pathologic complete response (pCR) on survival in retrospective studies, the inaccuracy of radiological modalities to adequately define the therapeutic response, and the poor interobserver agreement in defining baseline resectability status.^23^, ^24^, ^25^

### Preoperative therapy in PC: state of the art

Preoperative treatments, including chemotherapy and radiotherapy, have been investigated in the three different clinical scenarios of non-metastatic PC: borderline resectable, locally advanced unresectable, and resectable disease, as defined in Table 1.^26^, ^27^, ^28^, ^29^, ^30^, ^31^

**Table 1:** Criteria defining resectability status at diagnosis and associated standard treatments

Resectability status^a^	NCCN^31^	IAP consensus^30^	Standard treatment^b^
R	• No arterial tumour contact (CA, SMA, CHA). • No tumour contact with the SMV or PV or ≤180° contact without vein contour irregularity.	• SMA, CA, CHA: no tumour contact. • SMV/PV: no tumour contact or unilateral narrowing.	Surgery followed by adjuvant treatment. Consider staging laparoscopy and neoadjuvant therapy, particularly in high-risk patients.^c^
BR	*Pancreatic head/uncinate process:* • Solid tumour contact with CHA without extension to CA or hepatic artery bifurcation allowing for safe and complete resection and reconstruction. • Solid tumour contact with the SMA of ≤180°. • Solid tumour contact with variant arterial anatomy and the presence and degree of tumour contact should be noted if present, as it may affect surgical planning. *Pancreatic body/tail:* • Solid tumour contact with the CA of ≤180°. • Solid tumour contact with the CA of >180° without involvement of the aorta and with intact and uninvolved gastroduodenal artery thereby permitting a modified Appleby procedure (some panel members prefer these criteria to be in the locally advanced category). • Solid tumour contact with the SMV or PV of >180°, contact of ≤180° with contour irregularity of the vein or thrombosis of the vein but with suitable vessel proximal and distal to the site of involvement allowing for safe and complete resection and vein reconstruction. • Solid tumour contact with the IVC.	Subclassified according to SMV/PV involvement alone or arterial invasion. **BR-PV (SMV/PV involvement alone)** • SMV/PV: tumour contact 180° or greater or bilateral narrowing/occlusion, not exceeding the inferior border of the duodenum. • SMA, CA, CHA: no tumour contact/invasion. **BR-A (arterial involvement)** • SMA, CA: tumour contact of less than 180° without showing deformity/stenosis. • CHA: tumour contact without showing tumour contact of the PHA and/or CA. (Presence of variant arterial anatomy is not taken into consideration). Patients with anatomically resectable tumour and with performance status of 2 or more, CA 19-9 of >500 IU/ml and/or positive regional lymph node metastases.	Neoadjuvant therapy followed by surgery Consider staging laparoscopy
LA	Unreconstructible SMV/PV due to tumour involvement or occlusion (can be due to tumour or bland thrombus). *Head/uncinate process:* • Solid tumour contact with SMA >180°. • Solid tumour contact with the CA >180°. *Pancreatic body/tail:* • Solid tumour contact of >180° with the SMA or CA. • Solid tumour contact with the CA and aortic involvement.	• SMV/PV: bilateral narrowing/occlusion, exceeding the inferior border of the duodenum. • SMA, CA: tumour contact/invasion of 180° or more. • CHA: tumour contact/invasion showing tumour contact/invasion of the PHA and/or CA. • AO: tumour contact or invasion. • Macroscopic para aortic and extra abdominal lymph node metastasis (considered as metastatic disease).	Clinical trial (preferred). Induction chemotherapy (preferably 4-6 months) followed by chemoradiation or stereotactic body RT (SBRT) in selected patients (locally advanced without systemic metastases) or chemoradiation, or SBRT in selected patients who are not candidates for combination therapy.

Different classifications, based on the anatomic contact on imaging of tumour and blood vessel, have been proposed and adapted over time [MD Anderson Cancer Center (MDACC) guidelines,^30^ the Americas Hepato-Pancreato-Biliary Association/Society of Surgical Oncology/Society for Surgery of the Alimentary Tract (AHPBA/SSO/SSAT) expert consensus guidelines,^26^ the Intergroup Alliance,^27^ the International Study Group of Pancreatic Surgery (ISGPS) criteria,^28^ and NCCN guidelines^31^]. Recently, the consensus statement of the International Association of Pancreatology (IAP) added biological and conditional host-related factors to the classification based on imaging, including serum CA 19-9 of >500 IU/ml and/or positive regional lymph node metastases, and performance status of 2 or more.^29^

AO: aorta; BR: borderline resectable; CA: celiac axis; CHA: common hepatic artery; IVC: inferior vena cava; LA: locally advanced unresectable; PHA: proper hepatic artery; PV: portal vein; R; resectable; RT, radiotherapy; SMA: superior mesenteric artery; SMV: superior mesenteric vein.

a Decisions about resectability status should be made in consensus at multidisciplinary discussions.

b Participation in clinical trials is especially encouraged.

c High-risk patients: CA 19-9 more than 500 IU/ml, regional lymph node metastasis (biopsy or PET-CT), poor performance status (Eastern Cooperative Oncology Group score = 2, or more).

In borderline resectable PC the use of NAT has been associated with increased radical resection rates and superior overall survival in meta-analysis including cohort studies, retrospective observations, and phase I/II clinical trials.^32^^,^^33^ More recently, the first randomised phase III trial conducted in this setting (the Dutch PREOPANC trial), comparing preoperative chemoradiotherapy versus immediate surgery in patients with resectable or borderline resectable PC, showed that patients with borderline resectable tumours had significantly improved overall survival (OS), disease-free survival (DFS), and locoregional failure-free interval (LFFI) for preoperative chemoradiotherapy.^34^

In resectable disease the effectiveness of NAT is still uncertain, with conflicting results on survival benefit compared with upfront surgery.^3^^,^^35^ In one of the largest retrospective studies comparing NAT followed by resection and upfront resection, the NAT group was associated with improved survival compared with standard strategy [median survival, 26 months versus 21 months, respectively, hazard ratio (HR) 0.72; 95% confidence interval (CI), 0.68-0.78]. Patients in the upfront resected group had statistically significant higher pathologic T stage, positive lymph nodes, and positive resection margin. Compared with a subset of upfront resected patients who received adjuvant therapy, NAT patients had a better survival (median survival, 26 months vs 23 months, respectively, HR 0.83; 95% CI, 0.73-0.89).^18^ In addition, two recent systematic reviews and meta-analyses investigated the survival gain of NAT over standard treatment in patients with resectable tumour. Despite the significant improvement of radical resection rate, and the reduction of lymph nodes involvement, these studies did not show sufficient evidence for survival benefit of NAT when compared with upfront surgery (HR 0.96; 95% CI, 0.82-1.12^15^ and HR 0.86; 95% CI, 0.73-1.03^21^). However, in Lee et al., the subgroup of patients who completed NAT with subsequent resection had significantly increased survival than surgery followed by adjuvant treatment (HR 0.82; 95% CI, 0.71-0.93).^15^ Thus, a marginal favourable outcome in patients treated with NAT compared with those treated with the standard strategy may support this approach in resectable tumours. Despite these promising data, further randomised prospective studies are necessary to clearly establish the role of NAT in resectable PC.

The situation is different for patients with locally advanced unresectable tumours where systemic cytotoxic therapy is considered the first-choice treatment modality.^31^ Conversion surgery should be considered at multidisciplinary meetings and proposed in selected cases with optimal response after induction treatment, and only in specialised institutions. In a patient-level meta-analysis conducted on patients with locally advanced PC who underwent surgical resection after induction FOLFIRINOX, the percentage of conversion surgery ranged from 0% to 43% with a pooled percentage of 26% and an R0 rate between 50% and 100%.^36^ Due to conflicting results, it is still debatable whether patients should receive further ‘local regional’ therapy such as sequential chemoradiation or stereotactic body radiation therapy (SBRT) following induction chemotherapy.^37^^,^^38^

To summarise, current guidelines recommend NAT for borderline resectable PC, while upfront surgery followed by adjuvant treatment is still the standard recommendation for resectable disease except in cases that are high-risk for major abdominal surgery or in patients with high-risk characteristics (i.e. suspicion of advanced disease based on imaging findings or on significantly elevated carbohydrate antigen 19-9, large primary tumours or regional lymph nodes involvement, uncontrolled pain or excessive weight loss)^39^, ^40^, ^41^ (Table 1).

In locally advanced unresectable PC, primary systemic therapy constitutes the initial choice, and in some cases the addition of locoregional therapy can be considered for local control^36^^,^^42^^,^^43^ (Table 1).

Guidelines suggest the following options for preoperative treatment: FOLFIRINOX, modified FOLFIRINOX (m-FOLFIRINOX), gemcitabine, or gemcitabine/nab-paclitaxel.^39^, ^40^, ^41^^,^^44^ Multimodal treatment with chemoradiotherapy can be considered in selected cases, but the conclusions about its efficacy are controversial.^9^^,^^13^^,^^37^^,^^45^, ^46^, ^47^ Additional strategies such as perioperative treatments showed early promising results but need further investigation.^48^

Importantly, when preoperative therapy is indicated, current guidelines advice to refer patients to high-volume centres and encourage participation in clinical trials considering the limited evidence to recommend specific neoadjuvant regimens off-study.^39^

It is worth highlighting that the aforementioned recommendations on preoperative treatment in non-metastatic PC produced by the most important international cancer societies, are based on systematic reviews of cohort studies (Oxford Levels of Evidence category 2A) due to the lack of large phase III randomised controlled trials conducted in this setting.^39^, ^40^, ^41^^,^^44^ Additionally, results of published studies are often confounded by low patient numbers and lack of consensus regarding the definition of what precisely constitutes resectable, borderline resectable, and locally advanced—unresectable—disease.^49^ Thus, considering the overall lack of high-quality data from randomised controlled trials, several queries still need to be addressed such as the optimal candidates for preoperative treatment, the optimal treatment and number of therapeutic cycles, the timing of surgery after treatment, the additional benefit of sequential post-operative therapy (perioperative strategy), as well as the role of radiotherapy.^50^

### Clinical relevance of PC molecular subtyping

To date, all available evidence on preoperative treatment relies on studies with a ‘one-size-fits-all’ design, without the use of a prognostic or predictive biomarker-based selection process. This ‘all-comers’ approach has widely characterised the drug development process in PC and has been associated with a series of failures during the past 50 years, with only modest gain in survival obtained with polychemotherapy regimens in undefined patients subgroups.^51^ Recent insights from modern next-generation sequencing (NGS) technologies, shed light on the biological rationale of the disappointing results achieved so far. Indeed, PC is characterised by high molecular heterogeneity which results in different clinical behaviours among patients with similar tumour characteristics and presentation, including prognosis and treatment response/resistance.^52^, ^53^, ^54^ Over the last decade, several attempts to subtype PC based on commonly altered molecular networks have been made. This has led to the identification of subgroups based on genomic and transcriptomic analysis, sharing similar biological and clinical characteristics.

#### Genomic subtypes

**Figure 1:**
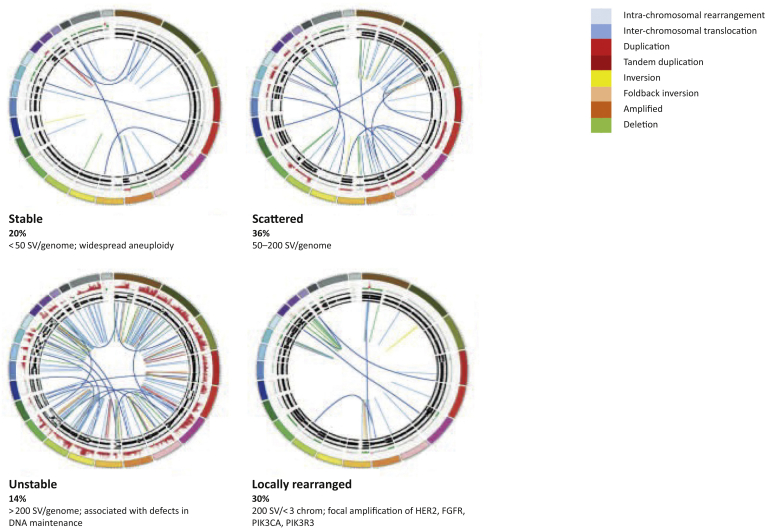
Whole-genome characterization of pancreatic cancer. Subtypes of pancreatic cancer based on the number and pattern of chromosomal structural variants (SV). The coloured outer rings are chromosomes, the next ring represents copy-number changes (red = gain, green = loss), the inner rings represents allele frequency. The inner lines represent chromosome structural rearrangements detected by whole-genome paired sequencing and the legend indicates the type of rearrangement. Reprinted by permission from Macmillan Publishers Ltd.: Nature 518:495-501, copyright 2015.

Whole-genome sequencing (WGS) allowed the classification of PC into four subtypes according to the frequency and distribution of structural variation of the genome: stable genomes (<50 structural variants per genome); scattered genomes (5-200 structural variants per genome); locally rearranged genomes (>200 structural variants clustered on <3 chromosomes); or unstable genomes (>200 structural variants distributed across the genome) (Figure 1).^53^ One of the most clinically meaningful subclasses resulting from this classification is represented by unstable tumours. Interestingly, in this group, a number of structural variants >558 was associated with significant defects in DNA damage response (DDR), particularly in homologous recombination repair (HRR) system. Additionally, genomic instability co-segregated with inactivation of DNA maintenance genes (*BRCA1*, *BRCA2*, or *PALB2*) and a mutational signature of DDR deficiency.^53^ Overall, alterations in DDR/HRR pathway were found in 24% of patients and were associated retrospectively with response to platinum-based chemotherapy.^53^ This finding had important clinical implications as it defined homologous recombination deficiency (HRD) as potential biomarker of therapeutic vulnerability to DNA damage agents, such as platinum and poly-ADP ribose polymerase inhibitors (PARPi).^55^, ^56^, ^57^, ^58^, ^59^, ^60^

Importantly, within HRD, germline mutations in *BRCA1* and *BRCA2* genes (which are the best characterised cause of HRD) have been associated with response to the PARP inhibitor olaparib in a phase III clinical trial (POLO) conducted in metastatic, platinum-sensitive, PC patients (first line setting).^61^ This was the first phase III trial that targeted a clinically relevant predictive biomarker in PC. This resulted in practice-changing governance with the approval of olaparib as maintenance strategy in platinum-sensitive advanced PC patients with *BRCA* germline mutations by the United States Food and Drug Administration (FDA) and European Medicines Agency (EMA). Several other trials are investigating PARPi in metastatic PC patients including those with germline and somatic mutations not only in *BRCA*, but also in other HRD genes.^59^^,^^60^

It has also been documented that a small proportion of PC (1%-2%) has defects in the DNA response process resulting from dysfunctions in DNA mismatch repair (MMR). These tumours demonstrate microsatellite instability (MSI), which can be reliably detected by routine immunohistochemical assays for MSH1, PMS2, MLH1, and MSH6 expression, and can potentially be treated with immune checkpoint blockade therapy.^62^

#### Transcriptomic subtypes

**Figure 2:**
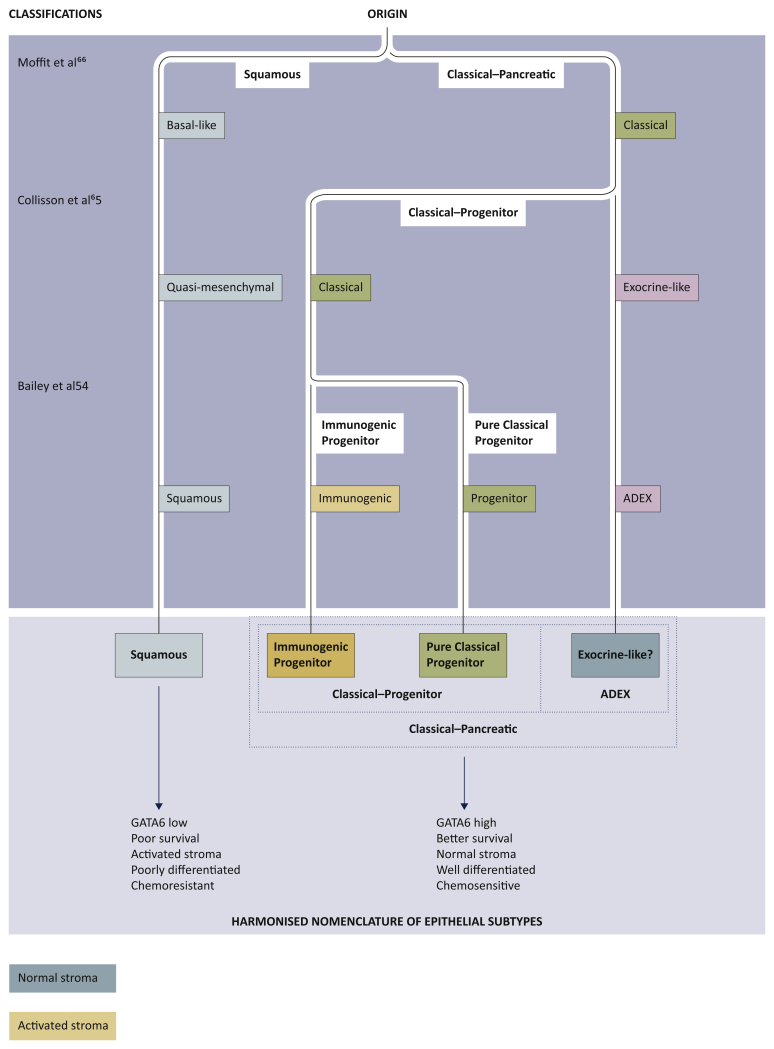
Phylotranscriptomic tree of pancreatic cancer. Two initial lineages are evident, largely driven by epigenetic events that separate pancreatic ductal adenocarcinoma into squamous (alternatively named basal-like and quasi-mesenchymal) and classical subtypes. The classical-pancreatic subtype might contain a spectrum of tumours that resemble pancreatic precursors, paralleling lineages occurring during pancreatic development. We can then discern a classical-progenitor subtype and, although it is unclear as to whether more differentiated progenitor subtypes are due to contamination by normal epithelium, an aberrantly differentiated endocrine exocrine (ADEX) subtype. Although the immunogenic subtype is largely driven by the immune infiltrate of the tumour microenvironment, epithelial-specific mechanisms probably exist that generate such an immune response. Stromal subtypes have also been discerned and, currently, do not appear to be directly associated with epithelial subtypes. The harmonised nomenclature has two broad subtypes: squamous and classical-pancreatic, with the classical-progenitor and ADEX subtypes residing in the latter. The classical-progenitor subtype further subdivides into the immunogenic progenitor and pure classical progenitor subtypes. Adapted from Collisson EA, Bailey P, Chang DK, Biankin AV. Molecular subtypes of pancreatic cancer. *Nat Rev Gastroenterol Hepatol* 2019; 16: 207-220,^64^ with permission from Springer Nature Limited. Copyright © 2019.

More recently, PC has been classified by multiple groups using associated transcriptional networks that proposed several different but overlapping classifications (Table 2 and Figure 2).^63^^,^^64^ Collisson et al. identified three molecular subtypes using hybridisation array-based mRNA expression: classical, quasi-mesenchymal (QM-PDA) and exocrine-like.^65^ The classical subtype expressed *GATA6* (the endodermal lineage-specifying transcription factor) and exhibited *KRAS* dependency while QM-PDA subtype correlated with high tumour grade and poor prognoses.^65^^,^^66^ Similarly, Moffitt et al., identified two tumour subtypes (basal-like and classical) and two stromal subtypes (normal and activated) as result of non-negative matrix factorisation (NMF) and virtual microdissection of microarray and RNAseq data from primary and metastatic PC tumours.^67^ This study showed that classical subtype was associated with better outcome compared with the basal one, instead characterised by worse survival and potentially larger benefit from adjuvant chemotherapy.

**Table 2:** Molecular subtyping of pancreatic cancer

Study	Histopathology and methodology	Subtypes	Biological insight	Clinical relevance
Moffitt et al.^67^	(*n* = 206; 145 primary PDAC and 61 metastatic PDAC) mRNA expression microarray (*n* = 206; 134 normal sites) and RNAseq in 15 primary samples, 37 PDXs, 3 cell lines, and 6 CAFs	Epithelial: Basal-like Classical Stromal: Activated Normal	• Different stromal subtypes may explain differences in stromal therapy observed in preclinical models • Metastases retain subtype signature • Basal-like subtype in majority of metastases • Lung metastases associated with classical subtype	• Poor survival in basal-like subtype and activated stroma in classical subtype • Basal-like subtype benefits from adjuvant chemotherapy • Stroma-targeted therapies might need to be subtype directed
Collisson et al.^65^	*n* = 85 primary untreated PDAC Microdissected (*n* = 27), whole PDAC (*n* = 39), and PDCLs (*n* = 19) Non-negative matrix factorization and consensus clustering	Classical Quasi-mesenchymal Exocrine-like	• Absence of exocrine-like subtype in ATCC PDAC cell lines • Subtype-specific function for GATA6 and KRAS addiction in classical subtype	• Poor survival for quasi-mesenchymal subtype, better for classical subtype • Quasi-mesenchymal subtype more sensitive to gemcitabine • Classical subtype • More sensitive to erlotinib
Bailey et al.^54^	*n* = 266 primary untreated PDAC Consensus clustering to subtypes according to signatures defined by Moffitt et al.^67^ and Collisson et al.^65^ RNAseq (*n* = 96) and expression array (*n* = 266)	Squamous Immunogenic Pancreatic progenitor ADEX	• Squamous subtype enriched for inflammation, metabolic reprogramming, cell proliferation, and epigenetic downregulation of endodermal genes • Squamous and immunogenic subtypes enriched for immune signalling including macrophages and T-cell subpopulations, respectively • Squamous subtype associated with adenosquamous histology; pancreatic progenitor associated with colloid and IPMN	• Poor survival in squamous subtype • Subtype-specific therapeutic targets including metabolic and cell cycle inhibitors and immunomodulation • Myeloid depletion in squamous subtype and immune evasion in immunogenic subtype

ADEX: aberrantly differentiated endocrine exocrine; ATCC: American Type Culture Collection; CAF: cancer-associated fibroblast; IPMN: intraductal papillary mucinous neoplasm; PDAC: pancreatic ductal adenocarcinoma; PDCL: patient-derived cell line; PDX: patient-derived xenograft; RNAseq: RNA sequencing; TCGA: The Cancer Genome Atlas.

Additionally, the Australian PC Genome Initiative (APGI; http://www.pancreaticcancer.net.au), as part of the International Cancer Genome Consortium (ICGC), defined four subtypes of PC through an integrated genomic analysis of transcriptomes, methylome, and mutational and histopathology data: squamous, pancreatic progenitor, immunogenic, and aberrantly differentiated endocrine exocrine (ADEX).^54^ In this study, the squamous subtype was enriched in gene programs typical of histologically squamous tumors of breast, bladder, lung, head and neck. These included biological pathways involved in inflammation, hypoxia response, metabolic programming, and TGF-β signalling.^68^ It overlapped with histopathologic adenosquamous tumours and was characterised by poor survival. Instead, the pancreatic progenitor subtype correlated with better outcome and expressed pathways involved in pancreatic endodermal differentiation. The ADEX group (a subclass of pancreatic progenitor tumours) was defined by transcriptional networks characterised by the simultaneous expression of transcriptional programs observed in the endocrine and exocrine pancreas, typically activated in the later stages of pancreatic development and differentiation.

Lastly, the immunogenic subtype, described by extending the analysis to the transcriptome of the immune infiltrate in the tumour microenvironment, was enriched for molecular signalling involved in immune cell infiltration and related immune response pathways.^54^

Despite discrepancies in nomenclatures and methods of identification, a substantial overlap exists between the different classifications with two main clinically relevant subgroups identified: squamous/basal-like and classical tumours (Figure 2). Squamous and basal-like (and QM-PDA) phenotypes share important aspects including the correlation with high tumour grade, metastatic disease, chemoresistance, and poor prognosis.^57^^,^^67^^,^^69^^,^^70^ On the other side, classical subtypes have generally a more favourable clinical outcome. These differences have also been documented in the recent genomics-driven COMPASS trial for advanced PC, which investigated the correlation between the therapeutic response to different treatment regimens and the transcriptomic profile obtained through tumour biopsy and RNA sequencing.^70^ The results of this study showed an overall response rate of 10% for basal-like and of 33% for classic tumours (*P* = 0.02).^70^ Notably, in patients treated with m-FOLFIRINOX, the progression rate was 60% in basal-like tumours compared with 15% in classic PC (*P* = 0.0002), with median OS of 5.9 months and 9.3 months for basal-like and classic, respectively (HR 0.47; 95% CI, 0.32-0.69, *P* = 0.0001).^70^ The expression of *GATA6* has been proposed as a surrogate biomarker for the differentiation between basal-like and classic subtype, based on the observation that basal-like tumours have significantly lower levels of *GATA6*.^57^^,^^69^^,^^70^ However, whether *GATA6*-low can be used as a predictive biomarker of therapeutic response needs further investigation.^57^^,^^70^^,^^71^

An additional clinically relevant PC subtype is the immunogenic, enriched with infiltrating cytotoxic CD8+ T cells, regulatory T and B cells, and high expression of cytotoxic T-lymphocyte-associated protein 4 (CTLA-4) and programmed cell death 1 (PD-1) immune checkpoint proteins. In tumours with these molecular characteristics, there is a biological rationale for the investigation of immune modulation with checkpoint inhibitors.^39^

### The rationale for a precision preoperative medicine approach

**Figure 3:**
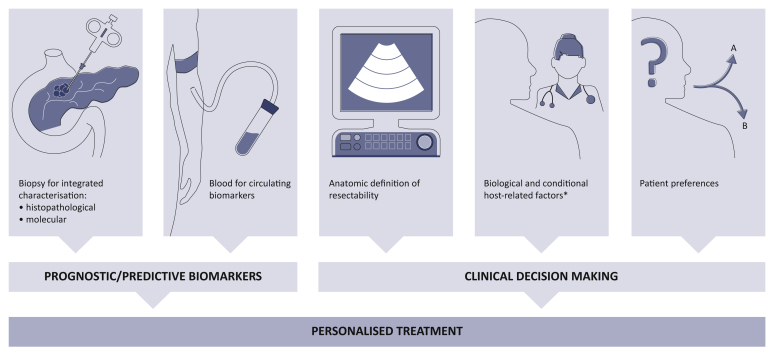
Ideal integration of clinical and biological information for personalised treatment of non-metastatic pancreatic cancer. The integration of clinical and pathological features with molecular data from biopsy specimens and blood tests should be used to investigate and validate prognostic/predictive models for personalised treatment selection. ∗Serum CA 19-9 of >500 IU/ml, positive regional lymph node metastases, performance status of two or more, comorbidities.

Considering the significant molecular heterogeneity of PC and the related prognostic and therapeutic implications, it is of utmost importance to implement biomarker-based preoperative clinical trials in order to validate prognostic and predictive factors, fundamental for an effective precision medicine approach. On one side, this would allow better risk stratification of candidates to upfront resection. On the other side, a genomic-driven precision approach may provide instruments useful for the choice of the optimal primary systemic treatment, by identifying biomarkers that could predict sensitivity or resistance to specific therapies. It is only through an integrated analysis of molecular prognostic/predictive biomarkers and clinical parameters that an individualised treatment path can be implemented in non-metastatic PC with the high potential to impact on therapeutic response, radical resection rate, and on survival (Figure 3).

As previously mentioned, the current decision algorithm in preoperative setting is predominantly based on imaging, clinical features, and blood tests and does not incorporate the tumour's biologic aggressiveness, chemoresistance, or metastatic propensity.^39^ Indeed, no biomarkers that predict treatment efficacy or resistance are currently available and robust prognostication models are still lacking. Recently, a preoperative prediction nomogram incorporating two biomarkers (S100A2 and S100A4), and clinical variables including age, tumour size, and location was developed and independently validated but its use in clinical practice is limited.^72^ Interestingly, preliminary results from a randomised phase II SWOG S1505 trial of perioperative m-FOLFIRINOX versus gemcitabine/nab-paclitaxel in resectable PC, showed similar results in terms of outcomes between the two therapeutic strategies thus indicating that in unselected populations it is almost impossible to see differences between different regimens as well as assess the relative role of platinum compounds versus other agents.^48^

It is therefore evident that precision medicine in non-metastatic PC remains an urgent and unmet need. Tailoring the therapeutic strategy on the molecular profile in preoperative setting is instead fundamental to improving outcome. Published studies reported exceptional responses after NAT, translating in long-term survival, in approximately 30% of patients,^73^, ^74^, ^75^, ^76^, ^77^, ^78^ while 17%-30% of cases progress during the therapy and up to 38% have no response.^32^^,^^79^ Progression during NAT likely reflects a more aggressive disease phenotype and has been empirically proposed as an indirect identifier of patients who will have limited benefit from curative surgery because of the high probability of relapse after resection.^80^ However, there is growing evidence that response to NAT is a crucial determinant of long-term prognosis and that primary chemoresistance reflects sub-optimal treatment for the majority of patients.^76^^,^^81^^,^^82^ The optimal treatment strategy for aggressive, chemotherapy-resistant tumours is challenging and needs to be defined. Data from a recent study showed that genetic or pharmacological depletion of histone methyltransferase enhancer of zeste homologue 2 (EZH2) can increase *GATA6* expression, thus inducing a subtype-switching in favour of a less aggressive, and potentially more therapy-susceptible, classical PC subtype.^83^ This may represent a promising strategy to be further investigated in squamous/basal-like tumours.

Alternatively, improvements in response rates and in clinical outcomes observed in exceptional and major responders are due to the effects of small subgroups of chemotherapy-sensitive patients.^32^^,^^84^ For example, we can speculate that the response rate reported in patients treated with platinum salts (approximately 30%),^14^^,^^85^ reflects a molecular background characterised by DDR/HRD (reported in up to 35% of early-stage PC patients) that has been associated with higher sensitivity to platinum-based chemotherapy.^53^^,^^55^, ^56^, ^57^^,^^86^ The identification of these subjects is important as the treatment with a platinum-backbone regimen would be more appropriate for those patients than gemcitabine-based chemotherapy.^58^ Furthermore, even in the presence of locally advanced unresectable tumours, the goal of treatment should be curative surgery in patients with HRD genome as treatment with a platinum-backbone regimen in these subjects is likely to result in higher response rates than gemcitabine-based chemotherapy, and is thus more likely to result in better outcomes and higher surgical resection rates.^53^^,^^58^ The ability to undergo tumour resection after primary systemic therapy is important as it constitutes the best chance of long-term survival for locally advanced PC compared with either no surgery or local procedures (HR 0.39; 95% CI, 0.34-0.46; *P* <  0.001).^87^ Thus, maximising the identification of likely platinum responders is fundamental considering the potentially significant impact on prognosis. However, whether HRD can predict response to platinum in early-stage disease needs further investigation as the majority of evidence derives from metastatic setting. It is indeed possible that different biological features between early-stage and advanced PC may result in different therapeutic susceptibility among tumours with similar molecular profile.^55^

**Figure 4:**
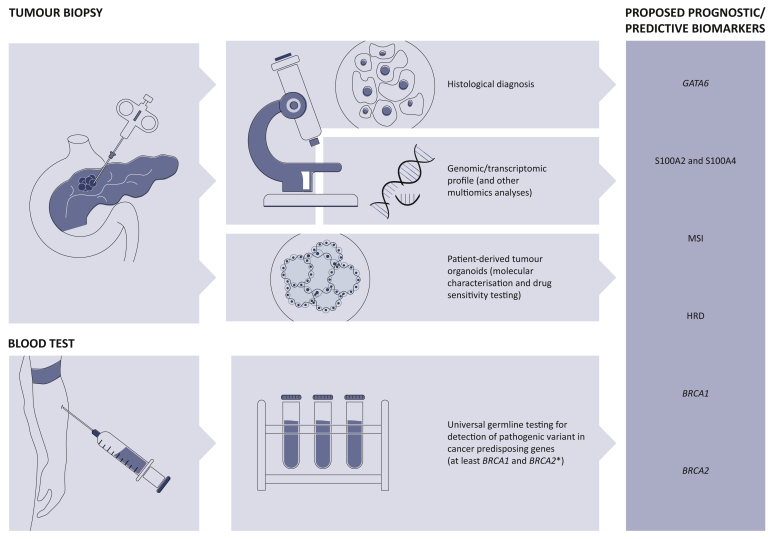
Proposed biomarkers to be implemented in future neoadjuvant clinical trials. HRD: homologous recombination deficiency, defined by germline or somatic mutations in HRD-related genes (*BRCA1/2*, *ATM*, *PALB2*, *FANC*-genes, *RAD51,* etc.), COSMIC3 signature, or genomic instability through structural variation patterns; MSI: microsatellite instability. ∗NCCN guidelines have been recently updated and recommend universal screening for germline variant in patients with PC, regardless of age, ethnicity, and family/personal history of cancer, including not only *BRCA1/2* but also *ATM*, *CDKN2A*, *PALB2*, *STK11*, *TP53*, *MLH1*, *MSH2*, *MSH6*, and *PMS2*.^31^

Several novel potential therapeutic targets are currently under investigation in metastatic PC and many others are on the horizon (Table 3).^88^ To date, the most clinically meaningful biomarkers that have potential to be successfully translated into the preoperative setting and incorporated in the design of future clinical trials are germline *BRCA1/2* mutations, HRD (more in general), and MSI. Prognostic biomarkers, such as S100A2/S100A4 and *GATA6* (to differentiate classical from squamous tumours), should be considered (Figure 4).

**Table 3:** Therapeutic targets in pancreatic cancer

Therapeutic target	Treatment	Study phase
***BRCA*, HRD**	PARPi, platinum	Phase I-III
**MMRd**	Immunotherapy^a^	Phase I, II
**HER2/HER3**	Zenocutuzumab	Phase I, II
**CDK4/6**	Palbociclib, PD-0332991	Phase I
**ALK**	Ceritinib	Phase I
**ERK1/2**	Ulixertinib, KO-947	Phase I
**TRK/ROS1**	Entrectinib, Larotrectinib	Phase II
**KRASG12C**	AMG 510	Phase I
**Metabolism**	Devimistat, hydroxychloroquine	Phase I-III
**TME**	PEGPH20, VCN-01, FAK/BTK inhibitors, ATRA	Phase I-III
BRAF	MAPK signalling inhibitors	
ATM	ATM inhibitors	
ATR	ATR inhibitors	
STK11	mTOR inhibitors	
FGFR	FGFR inhibitors	
Replication stress	ATR, WEE1 inhibitors	

Bold: Currently under clinical investigation

Roman: Preclinical evidence.

ALK, anaplastic lymphoma kinase; ATM, ataxia telangiectasia; ATR, ataxia telangiectasia and Rad3-related protein; ATRA, all-trans-retinoic-acid; BTK, Bruton's tyrosine kinase; FAK, Focal adhesion kinase; FGFR, fibroblast growth factor receptors; HRD, homologous recombination deficiency; MMRd, mismatch repair deficiency; PARPi, poly-ADP ribose polymerase inhibitors; PEGPH20, pegvorhyaluronidase-α; TME, extracellular tumour microenvironment.

a Immune checkpoint inhibitors, cancer vaccines, and CAR T-cell therapy.

### Challenges in implementing precision medicine in early-stage PC

The clinical relevance of preclinical data supporting a precision oncology approach needs to be validated in the clinic through biomarker-driven clinical trials. However, several hurdles limit implementation, including technical, organizational, and economic barriers (Table 4).^89^^,^^90^ There is concern about the ability of local pancreatic biopsy, especially using endoscopic ultrasound with fine needle aspiration (EUS-FNA), to obtain sufficient tissue for molecular profiling.^91^ Furthermore, low cellularity and abundant stroma, typical of PC biopsies, often hamper the possibility to perform additional analysis beyond cyto/histopathology.^92^ The highly spatial intratumoural heterogeneity of PC also prevents obtaining a reliable molecular characterization, representative of the entire tumour.^93^^,^^94^ In addition to these technical challenges, a few other interrelated factors hinder the successful clinical implementation of precision medicine in PC. Health systems face an overall lack of bioinformatics capacity specialised in the analysis and interpretation of complex data obtained from tumour sequencing. Furthermore, despite evidence of better outcomes for PC patients managed in high-volume centres,^95^^,^^96^ the vast majority are still diagnosed and treated at community hospitals where access to molecular analysis is limited and practising oncologists have little or no training to successfully use the information for clinical decision making.^89^ Conversely, the centralisation of current clinical implementation of multiomics technologies in highly specialised tertiary cancer centres poses important considerations about disparities of access to cutting-edge cancer programs. The situation is further complicated by the lack of biomarker-based clinical trials for PC patients, challenges in conducting adequately powered clinical trials in small molecular subgroups, the turnaround timing, costs/effectiveness, and reimbursement of molecular analyses. Recently, innovative therapeutic development platforms have been developed with the aim of integrating molecular data in clinical trials and accelerating precision therapeutic development for PC patients. These include PRECISION-Panc in the UK, EPPIC (Enhanced Pancreatic Cancer Profiling for Individualized Care) in Canada, and Precision Promise in the USA, which represent a possible solution to overcome the challenges mentioned earlier. These platforms aim to integrate discovery with preclinical development and innovative clinical trial design, allowing forward and backward translation.^73^^,^^97^ As part of PRECISION-Panc, to facilitate real world personalised clinical trials, a dynamic and flexible tissue acquisition and molecular profiling pathway has been developed (the PRECISION-Panc Master Protocol). This approach, based on extra passes on EUS pancreatic biopsy and peripheral venous sampling of blood for integrated multiomic analysis, delivers molecular profiling in patients with all stages of PC with a success rate of over 80%.^98^ The molecular information may guide eligibility for enrolment in a PRIMUS trial (pancreatic cancer individualised multi-arm umbrella study), investigating different biomarker-based treatment options.

A few other experiences have demonstrated the feasibility and the utility of molecular profiling in driving therapeutic choice in patients' metastatic PC, with positive impact on survival outcomes.^56^^,^^57^^,^^99^, ^100^, ^101^ It has been demonstrated that a molecular-driven precision medicine can be safely integrated into clinical management of PC patients with rapid turnaround time (<30 days).^56^^,^^57^^,^^99^, ^100^, ^101^ Thus, the incorporation of preclinical data for prognostic/predictive assessment in early-stage PC seems to be compatible with current standards. Indeed, the median waiting time from surgical consultation to surgery in high-volume centres is 29-31 days and potential delays in accessing surgery would seem not to negatively affect pathological features and survival of most patients.^102^^,^^103^ In addition, data from the US National Cancer Database (2003-2011), including 14 807 resected PC patients, indicate that an early allocation of surgery, within 12 weeks from diagnosis, is not associated with a survival benefit.^104^

### From hypothesis generation to clinical applicability

The next step is to advance this promising strategy in the preoperative setting, where precision medicine is still an unmet and urgent need. Amongst the emerging plethora of potential therapeutic vulnerabilities in PC, the most promising target is represented by the homologous recombination deficiency (HRD) pathway. The clinical relevance of this molecular characteristic in patients with early-stage PC has been recently pointed out in two retrospective studies. Golan et al. showed that patients with borderline resectable PC carrying germline *BRCA* mutations have an increased chance for pCR than wild type after neoadjuvant FOLFIRINOX (44.4% versus 10%, respectively; *P* = 0.009). Furthermore, the median OS after surgery was not reached among patients with germline mutations at 32 months for *BRCA* non-carriers (*P* = 0.2).^81^ This is consistent with other data reported in literature in which pCR was associated with better DFS and OS after surgery.^75^ Similarly, Yu et al. retrospectively studied patients with resected PC and a pathogenic germline mutation in *BRCA1*, *BRCA2*, and *PALB2*.^105^ Median OS in mutation carriers exposed to platinum in the perioperative setting was not reached versus 23.1 months in wild type patients (HR 0.12; 95% CI, 0.01-1.00). Patients in the mutation-positive group who received perioperative treatment with platinum had a trend toward improved median OS compared with those who did not (HR 0.15; 95% CI, 0.02-1.23; *P* = 0.07). Despite the retrospective design, these studies highlight the importance of a biomarker-driven treatment in the preoperative setting as it can guide the therapeutic choice in a personalised manner and can significantly improve patient outcomes. However, to the best of our knowledge, there are no prospective neoadjuvant clinical trials evaluating *BRCA* mutations as predictive biomarkers in PC (aside from locally advanced unresectable disease, which is usually included in clinical trials for advanced PC).

Currently, only a few trials are using a biomarker-enriched design in the neoadjuvant setting (summarised in Table 5). An ongoing prospective trial (PRIMUS002, NCT04176952) conducted in the context of PRECISION-Panc^97^ is investigating the potential predictive role of DNA damage repair (DDR) deficiency in patients treated with NAT. This is an integrated, open label, non-randomised, phase II study examining two therapeutic regimens (FOLFOX-A, i.e. 5-fluorouracil/leucovorin, oxaliplatin, nab-paclitaxel, and gemcitabine/nab-paclitaxel) given for 3 months before surgery in resectable and borderline resectable PC, aimed at assessing efficacy and toxicity with integrated translational work. Indeed, the study is powered to test a proposed DDR-deficient biomarker for response rate in patients treated with FOLFOX-A regimen. Particularly, this biomarker is a candidate HRD signature hypothesised to be a predictor of response to platinum-based therapy, and derived from a specific pattern of genomic structural rearrangements seen in known HRD cancers, from published and unpublished data sets.^73^^,^^106^ An additional phase II randomised study (PRIMUS-005, STAR-PAC2) will soon be activated and will investigate all-trans-retinoic-acid (ATRA) as a stromal targeting agent in a novel drug combination in locally advanced unresectable PC.^107^

**Table 4:** Barriers to implementation of precision medicine for pancreatic cancer

Sample-specific	Technology-specific	Practical/organisational	Therapeutic development
Tissue acquisition: • Difficult anatomy • Small-volume and heterogeneous nature of samples • Ethical considerations of repeated biopsies Tissue analysis: • Low cellularity • Abundant stroma • Intratumoural heterogeneity	Inconsistency in molecular test selection: • DNA: targeted-NGS, WES, WGS • RNA sequencing • IHC Challenges in: • Computational analysis • Data collection and storage • Data interpretation (actionability) • Integration between molecular information and clinical data • Data sharing and data mining	Timing of molecular testing: • Time to schedule biopsy • Turnaround times for molecular test results Geographical barriers to access precision medicine programs Lack of bioinformatic capacity Lack of provider awareness and education Lack of patient awareness Financial concerns: • Cost effectiveness • Reimbursement	Identification and validation of therapeutic targets Lack of specific molecular-targeted drugs Lack of biomarker-based clinical trials Challenges in conducting adequately powered clinical trials in small molecular subgroups Primary therapeutic resistance

IHC, immunohistochemistry; NGS, next-generation sequencing; WES, whole exome sequencing; WGS, whole-genome sequencing.

**Table 5:** Current biomarker-enriched preoperative clinical trials in pancreatic cancer

Trial ID	Biomarker	Therapeutic drugs	Phase	Status
NCT04176952	HRD signature	FOLFOX-A Gemcitabine/nab-paclitaxel	II non-randomised	Recruiting
NCT04005690	Multiple, not specified	Cobimetinib Olaparib	II non-randomised	Recruiting
NCT04481204	Multiple, not specified	Multiple drugs	II randomised	Not yet recruiting

Clinical trials including (not limited to) patients with resectable/borderline resectable pancreatic cancer. FOLFOX-A: 5-fluorouracil/leucovorin, oxaliplatin, nab-paclitaxel.

DDR, DNA damage response; HRD, homologous recombination system deficiency.

Another important biomarker-enriched study is investigating the association between mitogen-activated protein kinase inhibitor cobimetinib and PARP inhibitor olaparib in different clinical scenarios, including the NAT setting (NCT04005690). This is a phase II feasibility study in which validation of cobimetinib and olaparib molecular targets will be explored with tissue collection before and after therapy for biomarker evaluation. Several predictive biomarkers of therapeutic sensitivity/resistance are investigated; however, detailed information is not available from the study's description (https://clinicaltrials.gov/ct2/show/NCT04005690).

PARP inhibition is also being investigated in association with chemoradiotherapy in localised PC. The rationale for this approach is provided by preclinical studies, which showed remarkable synergy between radiotherapy and PARP1/2i veliparib in orthotopic animal models of non-metastatic PC.^108^ A recent phase I trial investigated safety and clinical efficacy of veliparib combined with gemcitabine-based chemoradiation in 30 locally advanced PC patients (NCT01908478) with translational analyses. The regimen was safe, tolerable, and clinically active.^109^ Median progression-free survival (PFS) and OS of the whole cohort were 9.8 months (95% CI: 8.4-18.6) and 14.6 months (95% CI: 11.6-21.8), respectively. Median OS was 19 months (95% CI: 6.2-27.2) in patients with impaired DDR tumours and 14 months (95% CI: 10.0-21.8) in patients with DDR proficient tumours. Expression of the DDR transcripts PARP3 and RBX1 were associated with improved OS.^109^ Despite the promising results showed in this study, further evidence is warranted to confirm the activity and efficacy of this multimodality strategy in potentially resectable patients.

Lastly, the PIONEER-Panc phase II randomised clinical trial (NCT04481204) will investigate novel therapeutic approaches in three clinical stage groups of localised PC based on Bayesian platform design. This trial entails exploratory translational multiomics analyses and organoids-based *in vitro* drug testing that will provide important information for the design of future biomarker-based phase III trials.

## Conclusion

The current knowledge on the molecular heterogeneity of PC poses important considerations about the future management of patients with non-metastatic disease. Firstly, the clinical investigation and validation of putative molecular prognostic biomarkers is imperative to identify the subset of patients who would benefit most from preoperative treatment rather than from upfront surgery. In parallel, it is fundamental to design genomic-driven clinical trials in order to test predictive biomarkers necessary to match the candidates to primary systemic therapy, tailored on the tumour molecular profile, thus allowing the opportunity for better treatment and survival outcomes. Furthermore, considering the relative rarity of non-metastatic disease, the possibility to significantly impact on the natural disease history with an optimal treatment strategy, and the surgical challenges on the definition of borderline resectable/resectable/locally advanced disease, it is advisable to refer these patients to high-volume centres with extensive expertise. Besides, due to the lack of high-quality data from randomised controlled trials, every candidate for preoperative treatments should be evaluated for enrolment in randomised (ideally molecularly-driven) clinical trials to guarantee the best therapeutic opportunity. Lastly, novel models of therapeutic development are warranted to investigate multiple hypothesis in small molecular subgroups to accelerate the drug testing process and approval and maximise the networking of centres with available clinical protocols, with possible referral to central high-volume institutes. It is only through major efforts in implementing a precision medicine approach that we can improve survival of PC patients.
